# Antibodies against p53 are associated with poor prognosis of colorectal cancer.

**DOI:** 10.1038/bjc.1995.386

**Published:** 1995-09

**Authors:** J. G. Houbiers, S. H. van der Burg, L. M. van de Watering, R. A. Tollenaar, A. Brand, C. J. van de Velde, C. J. Melief

**Affiliations:** Department of Immunohaematology and Blood Bank, University Hospital Leiden, The Netherlands.

## Abstract

**Images:**


British Journal of Cancer (1995) 72, 637-641

© 1995 Stockton Press All rights reserved 0007-0920/95 $12.00

### Antibodies against p53 are associated with poor prognosis of colorectal cancer

JGA Houbiers[1,2], SH van der Burg[1,2], LMG van de Watering[1], RAEM Tollenaar[2], A Brand[1], CJH van de Velde[2] and CJM Melief[1]

[1]*Department of Immunohaematology and Blood Bank and* [2]*Department of Surgery, University Hospital Leiden, PO Box 9600, 2300 RC Leiden, The Netherlands.*

**Summary** Mutation of the p53 gene is a common event in colorectal cancer. This alteration can result in cellular accumulation of p53 and may also induce p53 antibodies. Accumulation of p53 in tumour cells has been associated with poor prognosis of colorectal cancer. We tested preoperative sera from 255 patients with colorectal cancer by enzyme-linked immunosorbent assay (ELISA). A total of 70.2% had reactivity that was higher than the 'low' control serum. Employing a cut-off level of 10% of the 'high' control sample, 25.5% of the patients were positive for p53 antibodies. The presence of p53 antibodies correlated with the following prognostic factors: histological differentiation grade, shape of the tumour, and tumour invasion into blood vessels. Patients with p53 antibodies were shown to have decreased survival and decreased disease-free survival. Specifically for patients with cancer stage A and B1 the presence of p53 antibodies selected a subgroup with poor prognosis.

**Keywords:** colorectal cancer; p53

Colorectal cancer cells have accumulated several genetic alterations (Fearon and Vogelstein, 1990). The most common of these defects is loss of function of the tumour-suppressor gene p53 (Lane, 1992; Vogelstein and Kinzler, 1992). Although different mechanisms can result in loss of function of p53, allelic loss and mutation of the other gene is most often found (Baker *et al.*, 1989; Levine *et al.*, 1991; Vogelstein and Kinzler, 1992). These point mutations are associated with p53 overexpression caused by the decreased breakdown of the tetrameric form with mutant components (Levine *et al.*, 1991). The normal level of nuclear p53 expression is extremely low, but the aberrant accumulation of p53 in the tumour cell is detectable with p53-specific antibodies (Remvikos *et al.*, 1990; Bartek *et al.*, 1991; Lane, 1992). In colorectal cancer p53 is overexpressed in 50–70% of tumours (Cunningham *et al.*, 1992; Sun *et al.*, 1992; reviewed by Harris and Hollstein, 1993).

Because mutation in the p53 gene and the consequent overexpression of p53 are associated with tumour tissue, both, wild-type and mutant p53 may act as targets of tumour-specific humoral and cellular immune responses. p53-specific antibodies in the sera of patients have been studied in breast cancer (Crawford *et al.*, 1982; Davidoff *et al.*, 1992, Schlichtholz *et al.*, 1992; Mudenda *et al.*, 1994), in lung cancer (Winter *et al.*, 1992; Schlichtholz *et al.*, 1994), in B-cell lymphoma (Caron de Fromentel *et al.*, 1987) and more recently in various other types of cancer (Labrecque *et al.*, 1993; Lubin *et al.*, 1993; Angelopoulou *et al.*, 1994). In breast cancer, a p53 antibody response was found to be an indicator of poor prognosis (Schlichtholz *et al.*, 1992; Mudenda *et al.*, 1994).

Using an ELISA, we tested the serum samples taken preoperatively from 255 patients that participated in the CRAB clinical trial on the relationship between blood transfusion and colorectal cancer prognosis (Houbiers *et al.*, 1994), for the presence of p53 antibodies. The correlations between the presence of serum antibodies against p53 and prognostic factors of colorectal cancer and patient survival were investigated.

Correspondence: JGA Houbiers
Received 19 December 1994; revised 3 April 1995; accepted 21 April 1995

### Patients and methods

### *Patients*

Preoperative sera of 255 patients were tested for p53 antibodies. The colorectal cancer patients were enrolled in the CRAB clinical trial by seven different hospitals between 1987 and 1991 (Houbiers *et al.*, 1994). Data on patient history, surgery, tumour pathology, clinical complications and outcome during an average follow-up of 36 months were available. A total of 231 sera were drawn from patients operated upon with curative intent, while 24 sera were from patients with known metastatic disease at the time of surgery.

### *Assay*

The detection of p53 antibodies in patient sera was performed with a commercially available sandwich enzyme-linked immunosorbent assay (ELISA, p53-AK ELISA, cat. no. dia0301E, Dianova, Hamburg, Germany). The assay was performed according to the manufacturer's instructions and has the following specifications: 1:100 diluted patient serum was added for 60 min at 37°C to microtitre wells coated with recombinant p53. After washing, goat anti-human IgG antibody conjugated with peroxidase was added for 30 min at 37°C; finally the substrate 3, 5, 3, 5-tetramethylbenzidine (TMB) was added for 30 min. The enzymatic process was stopped by adding 2 N hydrogen chloride. Light absorption was measured at 450 nm on a photospectrometer (Titertek Multiskan).

This ELISA was validated by comparing the ELISA results of sera from cancer patients and non-cancer patients with results obtained from Western blotting with recombinant p53; the results of these sera showed a high concordance (H Zentgraf and PR Galle 1994, Heidelberg, personal communication). A total of 379 patients without tumours were negative in the p53 antibody assay (Müller *et al.* 1994).

The ELISA absorption data of our study were correlated with the manufacturer's control sera ('low' or 'high' concentration of p53 antibodies) by the following formula, which was designated the p53 antibody titre index (TI)

$$\text{p53-TI} = \frac{\text{patient sample absorption} - \text{absorption of low control}}{\text{absorption of high control} - \text{absorption of low control}} \times 100\%$$

The absorption results of sera from our own serum bank, added to control intertest variation showed good concordance between the different assays. Additionally, testing of the 'medium' control serum that was provided by the manufacturer resulted in p53-TIs in the narrow range from 31% to 35% in all assays. The sera of two patients that were excluded from the CRAB study because of a benign colon tumour had a p53-TI of ≤ 10%. The sera of 40 healthy blood donors also had a p53-TI of ≤ 10%.

*Immunohistochemistry*

A three step indirect immunoperoxidase technique on frozen 4-μm-thick colorectal carcinoma tissue sections was performed as described earlier (Ravenswaay Claasen Van *et al.*, 1992). The monoclonal antibodies PAb 122 (Gurney *et al.*, 1980), DO7 (Vojtesek *et al.*, 1992) and the polyclonal antiserum CM1 (Bartek *et al.*, 1991) were used to detect cellular accumulation (overexpression) of p53 and appeared concordant in all sections, except that PAb 122 stained more weakly and fewer cells were positive per section compared to the other two antibodies. From different parts of a resected tumour, on average five pieces of tissue were taken; if one or more pieces showed overexpression of p53 that tumour was considered positive for p53 accumulation. The results of immunohistochemical staining for p53 of colorectal carcinomas is further described elsewhere (Houbiers *et al.*, submitted).

*Analysis*

Using the Fisher's test for 2 × 2 tables (Fisher's exact test), the presence of p53 antibodies was correlated to the following prognostic factors of colorectal cancer: cancer stage [Dukes' classification modified according to Astler and Coller (1954)], histological differentiation, location of the tumour, tumour infiltration into adjacent organs, tumour size, tumour shape, tumour invasion of lymph vessels or blood vessels, sex, and a history of blood transfusion, blood groups (ABO, rhesus). Correlations between the presence of p53 antibodies and disease-free survival, overall survival, and risk of cancer recurrence were analysed using the Kaplan–Meier method and log-rank tests (for details, Houbiers *et al.*, 1994). Furthermore the association of p53 antibodies with the accumulation of p53 in tumour cells, as detected by immunohistochemistry on autologous tumour samples, was analysed with the Fisher's exact test.

### Results

Of the 255 tested preoperative sera from patients with colorectal cancer, 70.2% had a p53-antibody titre index (p53-TI) that was higher than the manufacturers 'low' control serum (i.e. >0%, Table I). When results of p53-TI ≤ 10% were regarded as absence of p53 antibodies, 25.5% of the sera were positive (Table I). The cut-off level of 10% was not arbitrarily chosen; samples with a lower absorption than the 'low control' that was provided by the manufacturer had a p53-TI between − 10% and 0%. Furthermore, the relevance of the 10% cut-off level was confirmed by the results of ten sera that were tested in an unmarked order for p53 antibodies using immunoblotting with recombinant p53 (Western blotting). The six sera that had a p53-TI ≤ 10% were negative by Western blotting (Figure 1); the two sera that appeared low or intermediate positive (+ or + +) by Western blotting scored intermediate positive by ELISA; the other two sera scored triple plus on the semiquantitative Western blot scale and had a p53-TI > 60% by ELISA.

The tumours of 20 of the 255 patients had been immunohistochemically stained for HLA antigens and cellular p53 accumulation (overexpression of p53). The association between tumours with p53 overexpression and serum p53 antibodies was significant: 7 out of the 12 patients with

**Table I** p53 antibodies in 255 colorectal cancer patients

| *p53-TI*[a] *(percentage of high control)* | *n* | *Percentage of patients* | *Cumulative percentage of patients* |
|---|---|---|---|
| ≤ 0 | 76 | 29.8 | 29.8 |
| 1– 10 | 114 | 44.7 | 74.5 |
| 11– 20 | 20 | 7.8 | 82.4 |
| 21– 30 | 12 | 4.7 | 87.1 |
| 31– 40 | 8 | 3.1 | 90.2 |
| 41– 50 | 1 | 0.4 | 90.6 |
| 51– 60 | 4 | 1.6 | 92.2 |
| 61– 70 | 7 | 2.7 | 94.9 |
| 71– 80 | 1 | 0.4 | 95.3 |
| 81– 90 | 1 | 0.4 | 95.7 |
| 91–100 | 0 | | |
| 100–110 | 4 | 1.6 | 97.3 |
| 110–120 | 0 | | |
| 120–130 | 2 | 0.8 | 98.0 |
| 130–140 | 2 | 0.8 | 98.8 |
| 140–150 | 0 | | |
| 150–160 | 1 | 0.4 | 99.2 |
| 160–170 | 1 | 0.4 | 99.6 |
| 170–180 | 1 | 0.4 | 100.0 |

[a]p53-TI, (patient sample - low control) (high control - low control) × 100%.

tumours showing p53 overexpression had a p53-TI > 10%, while only one out of the eight without p53 overexpression had antibodies ($P = 0.05$).

*Correlation of p53 antibodies with prognostic factors*

Table II gives a summary of the correlation of the p53-antibody status to known prognostic factors for colorectal cancer. The analyses with the cut-off level for the antibody-negative status on p53-TI ≤ 10% is shown. A significant association was found with histological grade, shape of the tumour, invasion of the tumour into blood vessels and the Quetelet index (i.e. the quantitative relation to the ideal body weight). Division of the p53-TI scale into two classes (≤ 0%, > 0%), three (< 10%, 10–33%, and > 33%) or into four (< 10%, 10–33%, 33–80%, > 80%) classes resulted in similar distributions.

The cancer stage was weakly associated with p53 antibodies: comparison of p53 antibody status of patients with cancer stage A or B to those with stage C resulted in 21% *vs* 31% p53 antibody positivity ($P = 0.09$); while the division of the p53-TI into four classes revealed an association with a $P$-value of 0.06.

No asssociation was found between p53 antibody status and any of the following factors: age, a history of pregnancy, history of blood transfusion, ABO blood group system, rhesus blood group system, sex, a history of cholecystectomy, diabetes, chronic aspecific lung disease, extension of the tumour into other organs, ulcerative tumour, presence of metastatic disease, lymph vessel involvement or size of the tumour.

*Follow-up and p53 antibodies*

Colorectal cancer prognosis was expressed in terms of overall survival, disease-free survival and disease-free period at 3 years of follow-up and was analysed on the basis of Kaplan–Meier curves as previously reported (Houbiers *et al.*, 1994). Associations between cancer prognosis and p53 antibodies were observed (Table III). We further analysed the association of p53 antibody status with prognosis within the subgroups of the major prognostic variable of colorectal cancer: the cancer stage. Within the subgroup of patients with cancer stage A (invasion limited to the mucosa or submucosa) or B1 (tumour progression into, but not through, the muscle layer of the large bowel), those who had induced p53 antibodies showed a significantly reduced survival. Analyses of the subgroups with cancer stage B2 and cancer stage C revealed no significant results.

**Figure 1** Western blot analysis was performed on ten patient sera to confirm the results of the ELISA. The recombinant p53 molecules (lane C) have run through the gel until the mark at the right-hand side and were labelled with anti-p53 antibody (p53). The sera with the numbers (No.) 2, 4, 7, 10, 11 and 13 can be considered negative by Western blot ( – ); in the ELISA they scored similarly negative (p53-TI ⩽ 10%). The sera numbered 3 and 17 appeared low or intermediate positive (+ or++) and scored intermediate positive by ELISA (p53-TI). The other two sera (1 and 5) scored +++ on the semiquantitative Western blot scale and had a p53-TI >60% by ELISA.

**Table II** p53 antibodies and prognostic factors of colorectal cancer

| Factor | *p53-TI (10)*[a] + | *p53-TI (10)*[a] – | P-*value*[b] |
|---|---|---|---|
| | Number of patients | | |
| Rhesus factor[1] | | | |
| Positive | 12 | 30 | 0.63 |
| Negative | 51 | 153 | |
| Cancer stage[2] | | | |
| A + B1 | 16 | 49 | 0.17 |
| B2 | 19 | 79 | |
| C | 27 | 59 | |
| Histological grade[3] | | | |
| Well differentiated | 4 | 15 | 0.02 |
| Moderately differentiated | 40 | 136 | |
| Poorly differentiated | 18 | 22 | |
| Shape of the tumours[4] | | | |
| Stalked or sessile | 16 | 72 | 0.04 |
| Flat or circular | 41 | 97 | |
| Invasion of blood vessels[5] | | | |
| No | 53 | 173 | 0.02 |
| Yes | 12 | 15 | |
| Quetelet index[6] | | | |
| ⩽21 | 7 | 40 | 0.01 |
| 22-26 | 32 | 70 | |
| ⩾27 | 15 | 19 | |

[a]p53 antibody status is positive ( + ) if p53-TI > 10%. [b]*P*-value of one-sided Fisher's exact test (2 × 2) or chi-square (2 × 3). Data missing from patients: 1,9; 2,6; 3,20; 4,29; 5,2 (blood vessels); 6,72.

### *Multivariate analysis*

Multivariate survival analysis (i.e. Cox regression analysis) was employed to establish the independent prognostic value of the p53 antibody status. In a predictive model with the variables cancer stage, extension of the tumour, histological grade, and tumour invasion into blood vessels and survival, disease-free survival or disease-free period as outcome variable, the p53-antibody status appeared not to add significantly to the model.

**Table III** p53 antibody status and colorectal cancer prognosis

| *Division on basis of p53 antibody status* (n) | *p53-TI (10)*[a] + (n) | *p53-TI (10)*[a] – (n) | P-*value* |
|---|---|---|---|
| All patients (249) | (63) | (186) | |
| Overall survival[b] | 61 | 68 | 0.35 |
| Disease-free survival[b] | 51 | 58 | 0.50 |
| Disease-free period[b] | 59 | 63 | 0.73 |
| Curative[c] surgery (226) | (58) | (168) | |
| Overall survival | 64 | 72 | 0.22 |
| Disease-free survival | 56 | 64 | 0.26 |
| Disease-free period | 64 | 70 | 0.42 |
| Cancer stage A or B1 (64) | (16) | (48) | |
| Overall survival | 75 | 88 | 0.04 |
| Disease-free survival | 69 | 81 | 0.04 |
| Disease-free period | 87 | 88 | 0.22 |

[a]p53 antibody status is positive ( + ) if p53-TI > 10%. [b]Percentage of patients alive or disease free at 3 years of follow-up based on Kaplan–Meier curves; *P*-value of log-rank test. [c]No evidence of remaining malignant tissue.

### Discussion

Using ELISA, we studied the presence of p53 antibodies in preoperatively collected serum of 255 patients with colorectal cancer. A total of 70.2% of the patients had reactivity above the 'low' control (Table I); when employing a cut-off of p53-TI ⩽ 10%, 25.5% of the patients were positive for p53 antibodies. The cut-off level of 10% was not arbitrarily chosen; sera with ELISA results below the 'low control' ranged from p53-TI – 10% to 0% and the six sera with p53-TI ⩽ 10% that additionally were tested with immunoblotting were negative for p53 antibodies.

The only study reporting on p53 antibodies in colorectal cancer patients used different assays and detected a positive antibody status in 13 patients (16%) out of 82 tested (Angelopoulou *et al*., 1994). That report, however, gives no clinical or pathological details on the patient group, nor does it correlate the results with prognosis.

The observed associations between p53 antibody positivity and factors related to poor prognosis of colorectal cancer suggest that intracellular p53 accumulation is not sufficient to elicit a humoral response, but that free p53 has to be presented to the immune system. Thus, p53 antibodies seem an expression of aggressive and extended tumours. We found that low differentiation grade, flat or circular tumour shape, tumour invasion into blood vessels, and lymph node metastases were associated with p53 antibodies. The correlation of the Quetelet index (i.e. the quantitative relation to the ideal body weight) and the induction of p53 antibodies is difficult to interpret (Table II). Cachectic patients may have a decreased immune reactivity, while overweight patients might notice symptoms of colorectal cancer only in a more advanced stage.

The correlation of p53 antibodies with prognostic factors of colorectal cancer is reflected in the correlation with survival rates when univariately analysed (Table III). When tested in a multivariate survival analysis, p53-antibody status appeared to be insignificant and could not add any predictive value to a model with the major prognostic factors. This analysis indicates that p53 antibody status is dependent on the other factors. Upon analysis of subgroups, p53 antibody status was only associated with the prognosis for patients with an early stage of the disease (i.e. cancer stage A or B1). For these patients, p53-antibody status may provide additional information. An advanced stage of colorectal cancer at time of diagnosis apparently represents aggressive tumours. In these cases p53 antibody status lacks prognostic value.

Schlichtholz *et al.* (1992) and Mudenda *et al.* (1994) found a correlation between the presence of p53 antibodies and histological grade of the tumour in breast cancer patients: no correlations were observed with cancer stage, tumour size, lymph node involvement, or risk of cancer recurrence. The only other study that investigated the correlation of p53 antibodies with patient prognosis or prognostic factors did not find such associations in a group of lung cancer patients (Winter *et al.*, 1992). In 1993, Winter *et al.*, reported on a significant association between improved survival of lung cancer and the presence of serum antibodies against autologous tumour cell protein extracts. Two of the 21 positive sera from these 36 tested patients appeared specific for p53. These data suggest that an anti-tumour immune response, represented by the detected antibodies, may affect tumour growth.

With one exception, no p53 antibodies were found in the serum of patients with a tumour that lacked immunohistochemically detectable overexpression of p53. This result is comparable to observations that only tumours with missense mutations of p53, resulting in p53 overexpression, elicit p53 antibodies (Davidoff *et al.*, 1992; Winter *et al.*, 1992; Lubin *et al.*, 1993). The origin of detectable p53 antibodies in serum from one patient with a primary tumour lacking p53 overexpression is unclear. This autoantibody induction may be due to the unusual presentation of large amounts of wild-type p53 from necrotic large tumours or metastases. It is also conceivable that the p53 gene is mutated and p53 – although the altered protein does not accumulate and thus is undetectable by immunohistochemistry – is presented to the immune system (T-helper cells) differently or in higher concentrations resulting in antibody induction.

Although mutations throughout the p53 gene can result in stabilisation and overexpression, the study by Davidoff *et al.* (1992) found that only tumours with complexes between p53 and a 70 kDa heat shock protein can elicit p53 antibodies. These complexes were primarily found in tumours with p53 mutations in exons 5 and 6, whereas tumours of patients without p53 antibodies exclusively displayed mutations in exons 7 and 8. This finding gives an explanation for the low frequency of p53 antibodies in patients with p53 overexpressing tumours.

The induction of p53 antibodies is an autoimmunisation process caused by the presentation of accumulated p53 to the immune system (Lubin *et al.*, 1993), which apparently has not become tolerant to this self-protein. The detection of IgG class p53 antibodies indicates that $CD4^+$ T-helper cells have been activated. These T cells are required to provide help for activation of cytotoxic T cells and may be used for p53-directed immunotherapy. Indeed, we have been able *in vitro* to induce cytotoxic T cells specific for wild-type and mutant p53 peptides (Houbiers *et al.*, 1993).

A protective humoral or cellular immune response, however, has not been established in the studied patients, since the presence of p53 antibodies was correlated with poor prognosis. Therefore, p53 antibodies must be considered a paraneoplastic phenomenon that can merely be used as a marker for bad prognosis of colorectal cancer. The presence of p53 antibodies may identify subgroups with poor prognosis among patients with an early stage of cancer that may need additional therapy. Its value for decisions in the management of colorectal cancer patients is still to be determined.

**Acknowledgements**
We thank Dr PR Galle (Department of Medicine, University of Heidelberg, Germany) for performing the Western blotting and Dr DP Lane (Department of Biochemistry, University of Dundee, UK) for providing the antibody CM1. We thank Dr Th Wobbes (Department of Surgery, University Hospital Nijmegen) for providing test sera, Dr J Hermans (Department of Medical Statistics, Leiden University) for statistical assistance and Drs GJ Fleuren (Department of Pathology, Leiden University) and AJA Bremers (Department of Surgery, University Hospital Leiden) for critically reading the manuscript.

### References

ANGELOPOULOU K, DIAMANDIS EP, SUTHERLAND DJA, KELLEN JA AND BUNTING PS. (1994). Prevalence of serum antibodies against the p53 tumor suppressor gene protein in various cancer. *Int. J. Cancer*, **58**, 480-487.

ASTLER VB AND COLLER FA. (1954). The prognostic significance of direct extension of carcinoma of the colon and rectum. *Ann. Surg.*, **139**, 846-852.

BAKER SJ, FEARON ER, NIGRO JM, HAMILTON SR, PREISINGER AC, JESSUP JM, TUINEN P VAN, LEDBETTER DH, BARKER DF, NAKAMURA Y, WHITE R AND VOGELSTEIN B. (1989). Chromosome 17 deletions and p53 gene mutations in colorectal carcinomas. *Science*, **244**, 217-221.

BARTEK J, BARTKOVA J, VOJTESEK B, STASKOVA Z, LUKAS J, REJTHAR A, KOVARIK J, MIDGLEY CA, GANNON JV AND LANE DP. (1991). Aberrant expression of the p53 oncoprotein is a common feature of a wide spectrum of human malignancies. *Oncogene*, **6**, 1699-1703.

CARON DE FROMENTEL C, MAY-LEVIN F, MOURIESSE H, LEMERLE J, CHANDRASEKARAN K AND MAY P. (1987). Presence of circulating antibodies against cellular protein p53 in notable proportion of children with B-cell lymphoma. *Int. J. Cancer*, **39**, 185-189.

CRAWFORD LV, PIM DC AND BULBROOK RD. (1982). Detection of antibodies against the cellular protein p53 in the sera from patients with breast cancer. *Int. J. Cancer*, **30**, 403-408.

CUNNINGHAM J, LUST JA, SCHAID DJ, BREN GD, CARPENTER HA, RIZZA E, KOVACK JS AND THIBODEAU SN. (1992). Expression of p53 and 17p allelic loss in colorectal carcinoma. *Cancer Res.*, **52**, 1974-1980.

DAVIDOFF AM, IGLEHART JD AND MARKS JR. (1992). Immune response to p53 is dependent upon p53 HSP70 complexes in breast cancers. *Proc. Natl Acad. Sci. USA*, **89**, 3439-3442.

FEARON ER AND VOGELSTEIN B. (1990). A genetic model for colorectal tumorigenesis. *Cell*, **61**, 759-767.

GURNEY EG, HARRISON RO AND FENNO J. (1980). Monoclonal antibodies against Simian virus 40T antigens: evidence for distinct subclasses of large T antigen and for similarities among conviral T antigens. *J. Virol.*, **34**, 752-763.

HARRIS CC AND HOLLSTEIN M. (1993). Clinical implications of the p53 tumor-suppressor gene. *N. Engl. J. Med.*, **329**, 1318-1327.

HOUBIERS JGA, NIJMAN HW, BURG SH VAN DER, DRIJFHOUT JW, KENEMANS P, VELDE CJH VAN DE, BRAND A, MOMBURG F, KAST WM AND MELIEF CJM. *In vitro* induction of human cytotoxic T lymphocyte responses against peptides of mutant and wild-type p53. *Eur. J. Immunol.*, **23**, 2072–2077.

HOUBIERS JGA, BRAND A, WATERING LMG VAN DE, HERMANS J, VERWEY PJM, BIJNEN AB, PAHLPLATZ P, EEFTINCK SCHATTENKERK M, WOBBES TH, VRIES JE, DE KLEMENTSCHITSCH P, MAAS AHM VAN DE, VELDE CJH AND VAN DE. (1994). Randomised controlled trial comparing transfusion leukocyte-depleted or buffy coat-depleted blood in surgery for colorectal cancer. *Lancet*, **344**, 573–578.

HOUBIERS JGA, TOLLENAAR RAEM, BURG SH VAN DER, KAPITEYIJN HW, BRAND A, VELDE CJH VAN DE, MELIEF CJM AND FLEUREN GH. (1995). Loss of HLA expression is associated with reduced expression of LFA3 but not with over expression of p53 (submitted).

LABRECQUE S, NAOR N, THOMSON D AND MATLASHEWSKI G. (1993). Analysis of the anti-p53 antibody response in cancer patients. *Cancer Res.*, **53**, 3468–3471.

LANE DP. (1992). P53, guardian of the genome. *Nature*, **358**, 15–16.

LEVINE AJ, MOMAND J AND FINLAY CA. (1991). The p53 tumour suppressor gene. *Nature*, **351**, 453–456.

LUBIN R, SCHLICHTHOLZ B, BENGOUFA D, ZALCMAN G, TRÉDANIEL J, HIRSCH A, CARON DE FROMENTEL C, PREUDHOMME C, FENAUX P, FOURNIER G, MANGIN P, LAURENT-PUIG P, PELLETIER G, SCHLUMBERGER M, DESGRANDCHAMPS F, LE DUC A, PEYRAT JP, JANIN N, BRESSAC B AND SOUSSI T. (1993). Analysis of p53 antibodies in patients with various cancers define B-cell epitopes of human p53: distribution on primary structure and exposure on protein surface. *Cancer Res.*, **53**, 5872–5876.

MUDENDA B, GREEN JA, GREEN B, JENKINS JR, ROBERTSON L, TARUNINA M AND LEINSTER SJ. (1994). The relationship between serum p53 autoantibodies and characteristics of human breast cancer. *Br. J. Cancer*, **69**, 1115–1119.

MÜLLER M, VOLKMANN M, ZENTGRAF H AND GALLE P. (1994). Clinical implications of the p53 tumor-suppressor gene (letter). *N. Engl. J. Med.*, **330**, 865.

RAVENSWAAY CLAASEN HH VAN, KLUIN PM AND FLEUREN GJ. (1992). Tumor infiltrating cells in human cancer: on the possible role of CD16 + macrophages in antitumor cytotoxicity. *Lab. Invest.*, **67**, 166–174.

REMVIKOS Y, LAURENT-PUIG P, SALMON RJ, FRELAT G, DUTRILLAUX B AND THOMAS G. (1990). Simultaneous monitoring of p53 protein and DNA content of colorectal adenocarcinoma by flow cytometry. *Int. J. Cancer*, **45**, 450–456.

SCHLICHTHOLZ B, LEGROS Y, GILLET D, GAILLARD C, MARTY M, LANE D, CALVO I AND SOUSSI T. (1992). The immune response to p53 in breast cancer patients is directed against immunodominant epitopes unrelated to the mutational hot spot. *Cancer Res.*, **52**, 6380–6384.

SCHLICHTHOLZ B, TREDANIEL J, LUBIN R, ZALCMAN G, HIRSCH A AND SOUSSI T. (1994). Analyses of p53 antibodies in sera of patients with lung carcinoma define immunodominant regions in the p53 protein. *Br. J. Cancer*, **69**, 809–816.

SUN XF, CARSTENSEN JM, ZHANG H, STAL O, WINGREN S, HATSCHEK T AND NORDENSKJOLD B. (1992). Prognostic significance of cytoplasmic p53 oncoprotein in colorectal adenocarcinoma. *Lancet*, **340**, 1369–1373.

VOGELSTEIN B AND KINZLER KW. (1992). P53 function and dysfunction (review). *Cell*, **70**, 523–526.

VOJTESEK B, BARTEK J, MIDGLEY CA AND LANE DP. (1992). An immunochemical analysis of the human nuclear phosphoprotein p53. New monoclonal antibodies and epitope mapping using recombinant p53. *J. Immunol. Methods*, **151**, 237–244.

WINTER SF, MINNA JD, JOHNSON BE, TAKAHASHI T, GAZDAR AF AND CARBONE DP. (1992). Development of antibodies against p53 in lung cancer patients appears to be dependent on the type of p53 mutation. *Cancer Res.*, **52**, 4168–4174.

WINTER SF, SEKIDO Y, MINNA JD, McINTIRE D, JOHNSON BE, GAZDAR AF AND CARBONE DP. (1993). Antibodies against autologous tumor cell proteins in patients with small-cell lung cancer–association with improved survival. *J. Natl Cancer Inst.*, **85**, 2012–2018.


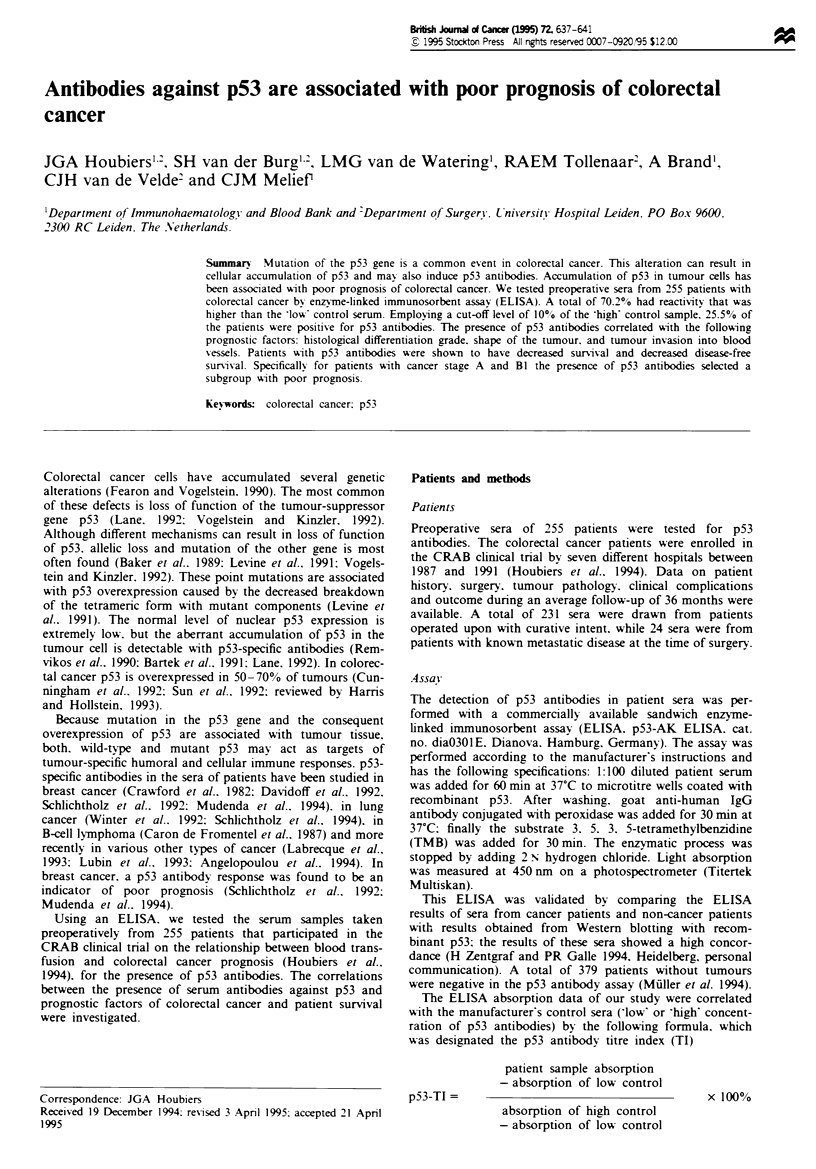

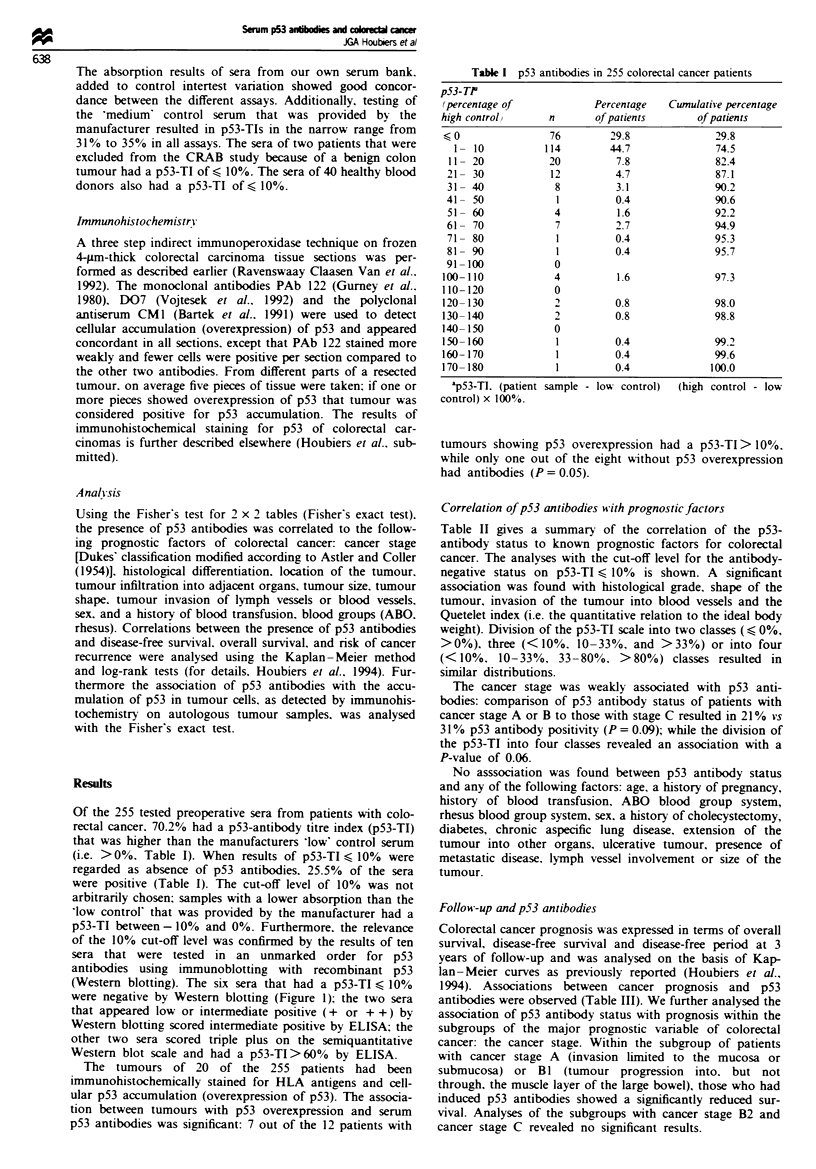

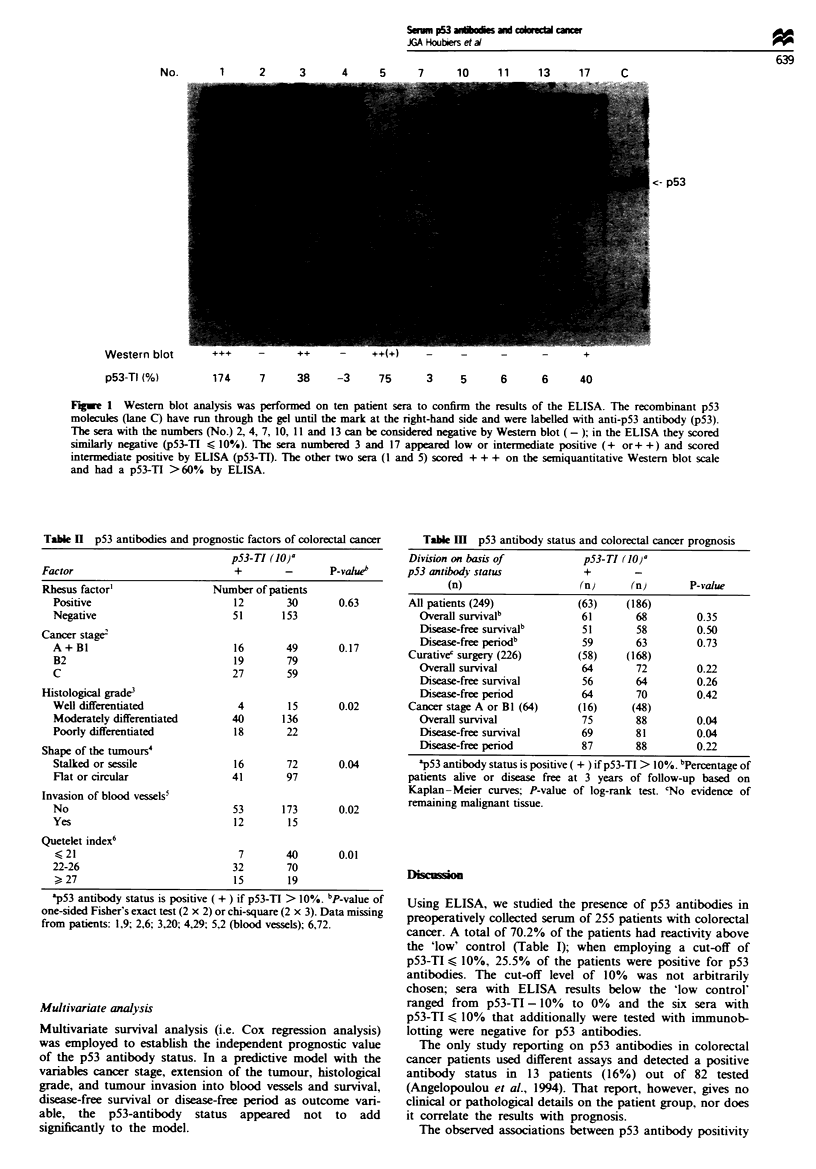

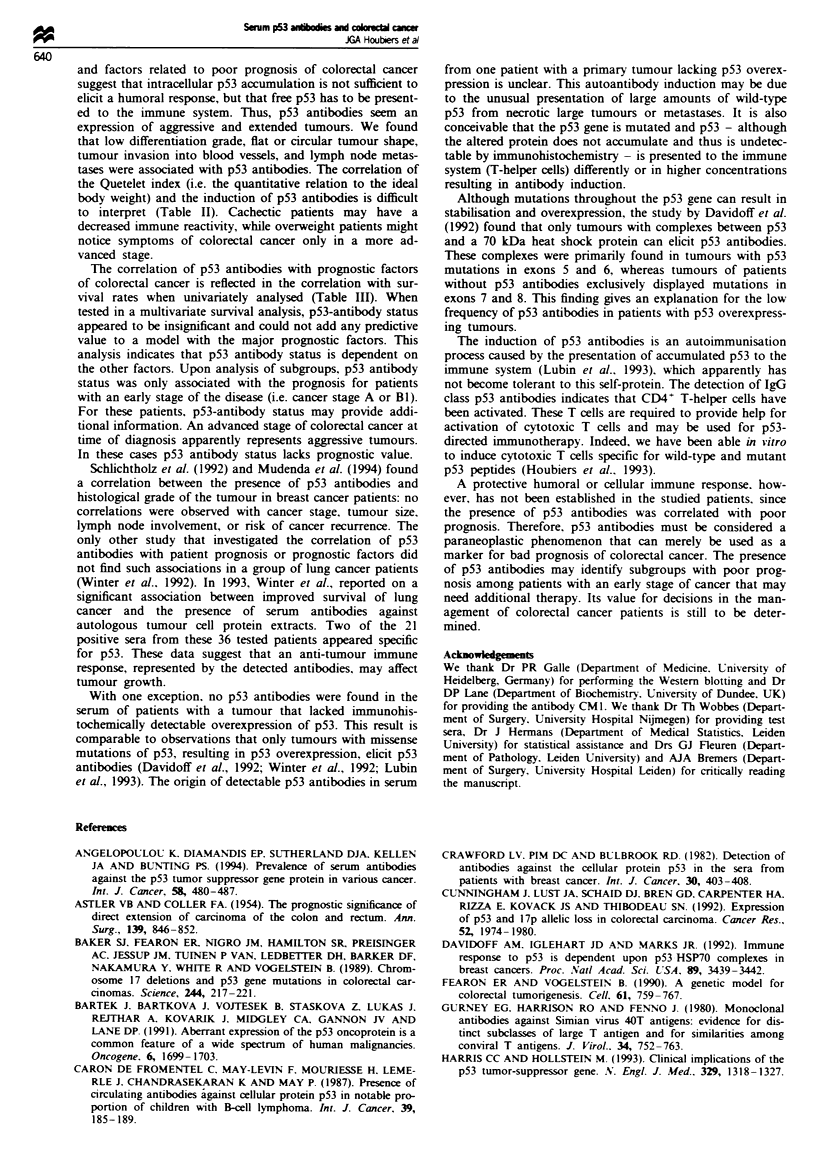

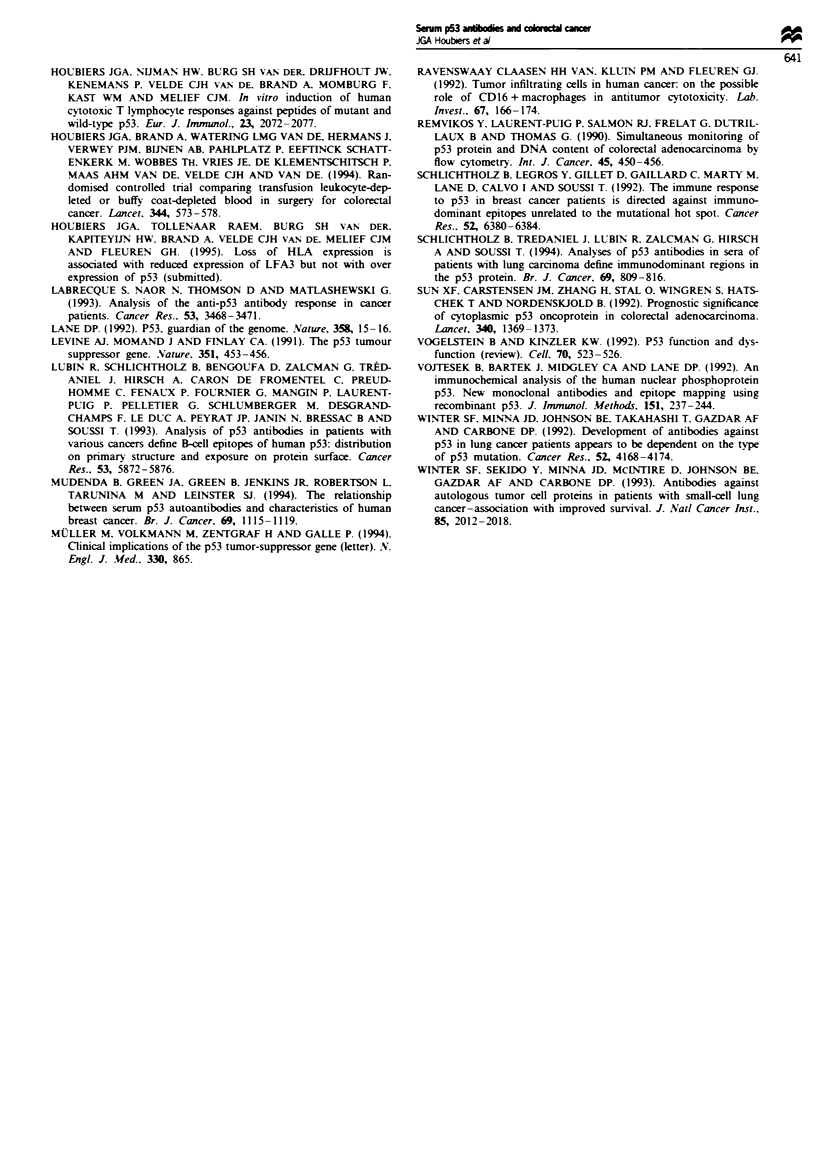


## References

[OCR_00543] ASTLER V. B., COLLER F. A. (1954). The prognostic significance of direct extension of carcinoma of the colon and rectum.. Ann Surg.

[OCR_00537] Angelopoulou K., Diamandis E. P., Sutherland D. J., Kellen J. A., Bunting P. S. (1994). Prevalence of serum antibodies against the p53 tumor suppressor gene protein in various cancers.. Int J Cancer.

[OCR_00546] Baker S. J., Fearon E. R., Nigro J. M., Hamilton S. R., Preisinger A. C., Jessup J. M., vanTuinen P., Ledbetter D. H., Barker D. F., Nakamura Y. (1989). Chromosome 17 deletions and p53 gene mutations in colorectal carcinomas.. Science.

[OCR_00553] Bártek J., Bártková J., Vojtesek B., Stasková Z., Lukás J., Rejthar A., Kovarík J., Midgley C. A., Gannon J. V., Lane D. P. (1991). Aberrant expression of the p53 oncoprotein is a common feature of a wide spectrum of human malignancies.. Oncogene.

[OCR_00562] Caron de Fromentel C., May-Levin F., Mouriesse H., Lemerle J., Chandrasekaran K., May P. (1987). Presence of circulating antibodies against cellular protein p53 in a notable proportion of children with B-cell lymphoma.. Int J Cancer.

[OCR_00567] Crawford L. V., Pim D. C., Bulbrook R. D. (1982). Detection of antibodies against the cellular protein p53 in sera from patients with breast cancer.. Int J Cancer.

[OCR_00575] Cunningham J., Lust J. A., Schaid D. J., Bren G. D., Carpenter H. A., Rizza E., Kovach J. S., Thibodeau S. N. (1992). Expression of p53 and 17p allelic loss in colorectal carcinoma.. Cancer Res.

[OCR_00580] Davidoff A. M., Iglehart J. D., Marks J. R. (1992). Immune response to p53 is dependent upon p53/HSP70 complexes in breast cancers.. Proc Natl Acad Sci U S A.

[OCR_00583] Fearon E. R., Vogelstein B. (1990). A genetic model for colorectal tumorigenesis.. Cell.

[OCR_00587] Gurney E. G., Harrison R. O., Fenno J. (1980). Monoclonal antibodies against simian virus 40 T antigens: evidence for distinct sublcasses of large T antigen and for similarities among nonviral T antigens.. J Virol.

[OCR_00610] Houbiers J. G., Brand A., van de Watering L. M., Hermans J., Verwey P. J., Bijnen A. B., Pahlplatz P., Eeftinck Schattenkerk M., Wobbes T., de Vries J. E. (1994). Randomised controlled trial comparing transfusion of leucocyte-depleted or buffy-coat-depleted blood in surgery for colorectal cancer.. Lancet.

[OCR_00605] Houbiers J. G., Nijman H. W., van der Burg S. H., Drijfhout J. W., Kenemans P., van de Velde C. J., Brand A., Momburg F., Kast W. M., Melief C. J. (1993). In vitro induction of human cytotoxic T lymphocyte responses against peptides of mutant and wild-type p53.. Eur J Immunol.

[OCR_00628] Labrecque S., Naor N., Thomson D., Matlashewski G. (1993). Analysis of the anti-p53 antibody response in cancer patients.. Cancer Res.

[OCR_00631] Lane D. P. (1992). Cancer. p53, guardian of the genome.. Nature.

[OCR_00634] Levine A. J., Momand J., Finlay C. A. (1991). The p53 tumour suppressor gene.. Nature.

[OCR_00636] Lubin R., Schlichtholz B., Bengoufa D., Zalcman G., Trédaniel J., Hirsch A., Caron de Fromentel C., Preudhomme C., Fenaux P., Fournier G. (1993). Analysis of p53 antibodies in patients with various cancers define B-cell epitopes of human p53: distribution on primary structure and exposure on protein surface.. Cancer Res.

[OCR_00649] Mudenda B., Green J. A., Green B., Jenkins J. R., Robertson L., Tarunina M., Leinster S. J. (1994). The relationship between serum p53 autoantibodies and characteristics of human breast cancer.. Br J Cancer.

[OCR_00653] Müller M., Volkmann M., Zentgraf H., Galle P. R. (1994). Clinical implications of the p53 tumor-suppressor gene.. N Engl J Med.

[OCR_00666] Remvikos Y., Laurent-Puig P., Salmon R. J., Frelat G., Dutrillaux B., Thomas G. (1990). Simultaneous monitoring of P53 protein and DNA content of colorectal adenocarcinomas by flow cytometry.. Int J Cancer.

[OCR_00673] Schlichtholz B., Legros Y., Gillet D., Gaillard C., Marty M., Lane D., Calvo F., Soussi T. (1992). The immune response to p53 in breast cancer patients is directed against immunodominant epitopes unrelated to the mutational hot spot.. Cancer Res.

[OCR_00677] Schlichtholz B., Trédaniel J., Lubin R., Zalcman G., Hirsch A., Soussi T. (1994). Analyses of p53 antibodies in sera of patients with lung carcinoma define immunodominant regions in the p53 protein.. Br J Cancer.

[OCR_00683] Sun X. F., Carstensen J. M., Zhang H., Stål O., Wingren S., Hatschek T., Nordenskjöld B. (1992). Prognostic significance of cytoplasmic p53 oncoprotein in colorectal adenocarcinoma.. Lancet.

[OCR_00689] Vogelstein B., Kinzler K. W. (1992). p53 function and dysfunction.. Cell.

[OCR_00695] Vojtesek B., Bártek J., Midgley C. A., Lane D. P. (1992). An immunochemical analysis of the human nuclear phosphoprotein p53. New monoclonal antibodies and epitope mapping using recombinant p53.. J Immunol Methods.

[OCR_00699] Winter S. F., Minna J. D., Johnson B. E., Takahashi T., Gazdar A. F., Carbone D. P. (1992). Development of antibodies against p53 in lung cancer patients appears to be dependent on the type of p53 mutation.. Cancer Res.

[OCR_00705] Winter S. F., Sekido Y., Minna J. D., McIntire D., Johnson B. E., Gazdar A. F., Carbone D. P. (1993). Antibodies against autologous tumor cell proteins in patients with small-cell lung cancer: association with improved survival.. J Natl Cancer Inst.

[OCR_00658] van Ravenswaay Claasen H. H., Kluin P. M., Fleuren G. J. (1992). Tumor infiltrating cells in human cancer. On the possible role of CD16+ macrophages in antitumor cytotoxicity.. Lab Invest.

